# Biochemical and Clinical Effects of Vitamin E Supplementation in Hungarian Smith-Lemli-Opitz Syndrome Patients

**DOI:** 10.3390/biom11081228

**Published:** 2021-08-17

**Authors:** Katalin Koczok, László Horváth, Zeljka Korade, Zoltán András Mezei, Gabriella P. Szabó, Ned A. Porter, Eszter Kovács, Károly Mirnics, István Balogh

**Affiliations:** 1 Division of Clinical Genetics, Department of Laboratory Medicine, Faculty of Medicine, University of Debrecen, 4032 Debrecen, Hungary; koczok@med.unideb.hu (K.K.); kovacs.eszter@med.unideb.hu (E.K.); 2 Department of Pharmaceutical Surveillance and Economics, Faculty of Pharmacy, University of Debrecen, 4032 Debrecen, Hungary; lhorvath@med.unideb.hu; 3 Department of Pediatrics, University of Nebraska Medical Center, Omaha, NE 68198, USA; zeljka.korade@unmc.edu; 4 Department of Laboratory Medicine, Faculty of Medicine, University of Debrecen, 4032 Debrecen, Hungary; mezeiza@med.unideb.hu; 5 Department of Pediatrics, Faculty of Medicine, University of Debrecen, 4032 Debrecen, Hungary; gabszamed@gmail.com; 6 Department of Chemistry, Vanderbilt University, Nashville, TN 37240, USA; n.porter@vanderbilt.edu; 7 Departments of Psychiatry, Biochemistry & Molecular Biology, Pharmacology & Experimental Neuroscience and Munroe-Meyer Institute for Genetics and Rehabilitation, University of Nebraska Medical Center, Omaha, NE 68106, USA; karoly.mirnics@unmc.edu; 8 Department of Human Genetics, Faculty of Medicine, University of Debrecen, 4032 Debrecen, Hungary

**Keywords:** Smith-Lemli-Opitz syndrome, vitamin E, vitamin A, behavioral disturbance, skin photosensitivity

## Abstract

Smith-Lemli-Opitz syndrome (SLOS) is a severe monogenic disorder resulting in low cholesterol and high 7-dehydrocholesterol (7-DHC) levels. 7-DHC-derived oxysterols likely contribute to disease pathophysiology, and thus antioxidant treatment might be beneficial because of high oxidative stress. In a three-year prospective study, we investigated the effects of vitamin E supplementation in six SLOS patients already receiving dietary cholesterol treatment. Plasma vitamin A and E concentrations were determined by the high-performance liquid chromatography (HPLC) method. At baseline, plasma 7-DHC, 8-dehydrocholesterol (8-DHC) and cholesterol levels were determined by liquid chromatography–tandem mass spectrometry (LC-MS/MS) method. The clinical effect of the supplementation was assessed by performing structured parental interviews. At baseline, patients were characterized by low or low–normal plasma vitamin E concentrations (7.19–15.68 μmol/L), while vitamin A concentrations were found to be normal or high (1.26–2.68 μmol/L). Vitamin E supplementation resulted in correction or significant elevation of plasma vitamin E concentration in all patients. We observed reduced aggression, self-injury, irritability, hyperactivity, attention deficit, repetitive behavior, sleep disturbance, skin photosensitivity and/or eczema in 3/6 patients, with notable individual variability. Clinical response to therapy was associated with a low baseline 7-DHC + 8-DHC/cholesterol ratio (0.2–0.4). We suggest that determination of vitamin E status is important in SLOS patients. Supplementation of vitamin E should be considered and might be beneficial.

## 1. Introduction

Smith-Lemli-Opitz syndrome (SLOS, OMIM #270400), an inborn error of cholesterol biosynthesis, is caused by reduced 7-dehydrocholesterol reductase (DHCR7) activity [1,2,3]. The inheritance pattern is autosomal recessive, and to date, 207 mutations have been reported in the encoding *DHCR7* gene (11q13.4) (Professional Human Gene Mutation Database, 2021.2 release). Due to DHCR7 deficiency, cholesterol synthesis is impaired, while 7-dehydrocholesterol (7-DHC) and its isomer, 8-dehydrocholesterol (8-DHC), are markedly elevated in plasma and tissues of SLOS patients [2,4,5]. Clinically, SLOS is most commonly associated with growth retardation, developmental delay, intellectual disability, multiple congenital anomalies, distinctive facial features and behavioral abnormalities, including severe sleep disturbance [6,7]. SLOS-associated abnormal behavior represents major challenges for the affected families and greatly influences quality of life [8]. It appears that cholesterol deficiency is not the only pathophysiological mechanism leading to SLOS phenotype. Rather, accumulation of 7-DHC and 8-DHC and their derivatives are all likely major contributors to disease pathophysiology [9]. However, present-day therapeutic interventions are primarily aimed toward normalizing cholesterol levels, mainly by dietary cholesterol supplementation, which is considered standard-of-care in SLOS. Yet, cholesterol supplementation alone, or in combination with statins (3-hydroxy-3-methylglutaryl coenzyme A reductase inhibitors), has not been proven effective in alleviating the numerous SLOS-associated behavioral challenges [10]. Wassif et al. reported reduced irritability and a significant improvement in serum dehydrocholesterol/total sterol ratio along with decreased cerebrospinal fluid dehydrocholesterol levels in SLOS patients on combined cholesterol and simvastatin therapy [11]. 

Previously, we have shown that 7-DHC is highly oxidizable and free radical peroxidation leads to the production of 7-DHC-derived oxysterols in in vitro and in vivo transgenic mouse models [12,13]. Considering the adverse effects of 7-DHC-derived oxysterols [14] and the positive effects of antioxidants in human dermal fibroblasts from SLOS patients and a transgenic mouse model [12], preventing the formation of 7-DHC oxysterols may counter the detrimental effects of *DHCR7* mutations. In addition to our studies, Fliesler et al. reported that a cholesterol-rich diet augmented with antioxidants (i.e., high levels of vitamins E and C plus sodium selenite) performs better in preserving retinal structure and function and lowering 7-DHC-derived oxysterol levels in a SLOS rat model compared to a cholesterol-rich diet alone [15].

The first clinical trial investigating the effects of antioxidant therapy is currently being undertaken at Children’s Hospital Colorado (https://clinicaltrials.gov/ct2/show/NCT01773278, accessed on 13 July 2021). Preliminary results suggest that combination therapy of cholesterol and AquADEKs^®^ (a commercial multivitamin and mineral preparation enabling enhanced absorption of fat-soluble vitamins) improves retinal function in SLOS patients (Elias, E., Braverman, R, Tong, S. Beyond cholesterol: Antioxidant treatment for patients with Smith-Lemli-Opitz syndrome, Abstract, Annual Meeting, American Society for Human Genetics. 2012, San Francisco, CA, USA). 

In a parallel, but unrelated study, in 2013, we started a three-year prospective study of vitamin E supplementation of patients with SLOS. After determining their baseline vitamin A and E status, we investigated the absorption, tolerability, side effects and behavioral changes in response to vitamin E supplementation.

## 2. Materials and Methods

### 2.1. Study Design

**Figure 1:**
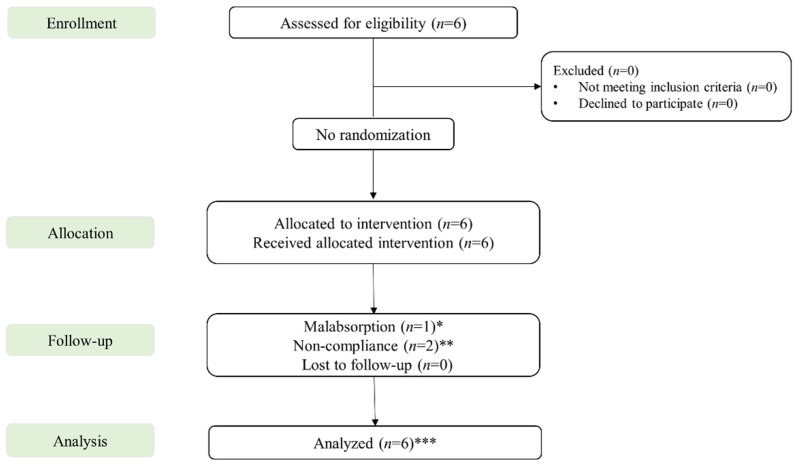
Flowchart for the single-case experimental study. * One patient developed daily, severe diarrhea after the first 1.5 years of the study, resulting in malabsorption of the supplement. ** In the case of two patients, vitamin E intake was irregular and inappropriate because of frequent hospitalizations. *** Data of all of the enrolled patients were analyzed.

We conducted a single-case experimental study (flowchart is shown in Figure 1) with a bi-phasic multiple participant AB design (A—baseline control/comparison phase, B—intervention phase) with no randomization or replication of the baseline or intervention phases [16].

Enrollment: inclusion criteria were clinical symptoms of SLOS plus genetic (two causative *DHCR7* gene mutations in trans) and/or biochemical (i.e., elevated serum 7-DHC level, since in the case of one patient only one mutation could be identified) confirmation of Smith-Lemli-Opitz syndrome (for detailed description see Section 2.2).

In the baseline control/comparison phase, plasma vitamin A/E (for a detailed description see Section 2.4.1), 7-DHC, 8-DHC and cholesterol (for a detailed description see Section 2.4.2, Section 2.4.3 and Section 2.4.4) concentrations were determined. 

In the intervention phase, patients received an oil-based, liquid vitamin E formulation (for a detailed description see Section 2.3). It has to be noted that in addition to the vitamin in the case of five patients, cholesterol supplementation, and in the case of one patient (Patient 6) both cholesterol supplementation and statin therapy, were continued as previously prescribed by the patients’ pediatricians during the entire study. 

Replications (demonstration of biochemical effects/changes): during the study, plasma vitamin A/E concentrations were monitored monthly during the first two months of supplementation. Subsequently, monitoring periods of 3–5-months proved to be sufficient and safe. There were 10–12 blood samples/patient tested. 

Replications (demonstration of clinical effects/changes): structured parental interviews (detailed description: Section 2.4.5) were performed two times during the study, after the first year and at the end of the study, in order to assess clinical effects of vitamin E supplementation. In the case of fluctuating plasma vitamin E levels (Patients 1, 2 and 4), interviews were performed during the time when the plasma vitamin E level was normal, i.e., only after the first year.

The primary outcome was to investigate the cause of the vitamin E deficiency in SLOS patients. As a secondary outcome, we aimed to assess behavioral changes in response to vitamin E supplementation.

Based on our preliminary data showing vitamin E deficiency in our patients (data not shown) and the presumed beneficiary effect of vitamin E proven in previous in vitro/in vivo studies [12,13], we decided not to give a placebo to any of the patients, therefore there were no control subjects.

### 2.2. Patients

Six Hungarian SLOS patients (4–21 years of age; female to male ratio 1:5) were enrolled in the study in 2013. Clinical presentation of the patients ranged from mild to classical. Serum 7-DHC levels were markedly elevated in all subjects, ranging from 87 mg/L to 302 mg/L (reference range 0.1 ± 0.05 mg/L for children < 10 years; 0.13 ± 0.06 mg/L for ≥10 years of age [9]), and cholesterol concentrations were 0.72–2.77 mmol/L (low in 4 out of 6 patients). 7-DHC and cholesterol measurements were performed previously in our laboratory before cholesterol or statin therapy were started [17,18]. 

*DHCR7* gene sequencing was also previously carried out in all patients and *DHCR7* mutations were found on both alleles in all but one of the enrolled patients [17,18]. 

### 2.3. Vitamin E Formula and Supplementation Regimen

An oil-based, liquid vitamin E formulation containing *all-rac*-α-tocopheryl acetate as the active ingredient (vehicle: oleum helianthi) was prepared by a clinical pharmacist. Based on our request, the National Institute of Pharmacy and Nutrition has authorized the use of tocopheryl in compounding and has put it on the “list of permitted active pharmaceutical ingredients used in compounding” in order to provide appropriate and individualized vitamin E supplementation for SLOS patients (permission number: GYEMSZI-OGYI 2/2012 MAG). 

Starting daily doses were 230 mg for patients aged 4–10 years old and 2 × 230 mg over 10 years of age. The supplement was administered with meals through gastrostomy tube or per os: 230 mg of *all-rac*-α-tocopheryl acetate is equivalent to 103.5 mg of α-tocopherol [19]. 

Based on plasma vitamin E concentrations measured during the first 12 months of supplementation, starting doses were decreased by 50% in the case of patients 3 and 6. In addition, but very rarely needed, short-term modifications on an empirical basis were also applied, e.g., in the case of elevated plasma vitamin E levels (above the upper limit of the reference range), the supplement was administered for only six days a week until the next follow-up. 

### 2.4. Methods

#### 2.4.1. Measurement of Plasma Vitamin A and E Concentrations

Whole blood was collected from non-fasting subjects into ethylene-diamine-tetra-acetic acid (EDTA) tubes, transported light-protected to the laboratory within 24 h after collection, and plasma was separated, frozen and kept at −20 °C until analysis. Plasma vitamin A and E concentrations were determined by the high-performance liquid chromatography (HPLC) method on the Jasco HPLC system (ABLE Jasco, Tokyo, Japan) using a commercially available in vitro diagnostic (IVD) kit, Vitamin A/E by HPLC (Bio-Rad Laboratories, Hercules, CA, USA). Measurements were carried out according to the manufacturer’s instructions in an isocratic system. Samples were separated on a reversed phase cartridge with subsequent UV detection at 305 nm (per the manufacturer’s recommendation for single-wavelength detection of vitamins A and E). Quantification was performed using an internal standard and calibrators provided by the kit manufacturer. 

Reference intervals (RI) applied for the evaluation are as follows: (1) plasma vitamin E concentration: 14.5–33.0 µmol/L (1–<19 years of age) [20] and 11.6–41.76 µmol/L (adults) [21], and (2) plasma vitamin A concentration: 1.0–1.6 µmol/L (1–<11 years of age) [20], 0.9–1.9 µmol/L (11–<16 years of age) [20] and 1.05–2.79 µmol/L (adults) [21]. 

On SLOS patients’ chromatograms, there were two additional unknown peaks observed compared to control plasma samples during HPLC analysis. The peak of vitamin E in SLOS samples was identified according to the retention time of vitamin E of the calibrator and spike-in experiments were also performed. Different amounts of the calibrator (25%, 50% and 75%) of known vitamin E concentration were added to SLOS plasma samples and vitamin E concentration of the mixtures was subsequently determined. Based on the high correlation between calculated and observed vitamin E concentrations (data not shown), we believe that the measured vitamin E concentrations are accurate in our SLOS patient samples. Thus, it was concluded that the additional peaks, observed from patients’ chromatograms, were unrelated to vitamin E treatment. 

It should be noted in passing that 7-DHC can interfere with vitamin E analysis and spuriously elevated levels of the vitamin are found under some chromatography conditions [22,23]. Under the HPLC method we used, an impurity readily separates from the vitamin, and we are confident that any interfering effects of 7-DHC have been removed with our HPLC conditions. 

#### 2.4.2. Sterol Analysis

Unless otherwise noted, all chemicals were purchased from Sigma-Aldrich Co. (St. Louis, MO, USA). HPLC-grade solvents were purchased from Thermo Fisher Scientific Inc (Waltham, MA, USA). [25,26,26,26,27,27,27-*d_7_*] 7-DHC was obtained by chemical synthesis as previously described [24,25]. 

#### 2.4.3. Lipid Extraction

Ten µL of human plasma was added to 800 μL of Folch solution containing 0.25 mg/mL TPP, 0.005% BHT and the internal standards *d_7_*-7-DHC (13 ng), ^13^C_3_-Des (100 ng), ^13^C_3_-Lano (100 ng) and *d_7_*-Chol (34 ng), followed by the addition of 400 μL of 0.9% NaCl. The resulting mixture was vortexed and centrifuged. The lower organic phase was recovered and dried under a stream of nitrogen, and 200 μL of 1 mg/mL of freshly prepared PTAD solution in MeOH was added to the residues of plasma extracts, and the solutions were incubated for 30 min at room temperature with occasional shaking and transferred into sample vials. The samples were stored at −80 °C until analysis by liquid chromatography–tandem mass spectrometry (LC-MS/MS).

#### 2.4.4. LC-MS/MS Conditions

LC separations were performed on a Waters Acquity Ultra Performance Liquid Chromatography (UPLC) system equipped with an autosampler (Waters, Milford, MA, USA) using a Waters Acquity UPLC (Ethylene Bridged Hybrid (BEH) C18 column (1.7 μm, 2.1 mm × 50 mm). A Triple stage quadrupole (TSQ™) Quantum ultra-tandem mass spectrometer (ThermoFisher, Waltham, MA, USA) was used for MS detections, and data were acquired with the Finnigan Xcalibur software package.

Analyses of 4-Phenyl-1,2,4-triazoline-3,5-dione (PTAD) derivatized samples were carried out with an isocratic solvent of methanol/0.1% acetic acid at a flow rate of 0.5 mL/min. MS/MS analysis of the PTAD derivatives was acquired in the positive ion mode using atmospheric pressure chemical ionization (APCI) and selected reaction monitoring (SRM). MS parameters were optimized for 7-DHC-PTAD and were as follows: auxiliary nitrogen gas pressure at 55 psi and sheath gas pressure at 60 psi, and discharge current at 22 μA and vaporizer temperature at 265 °C. Collision-induced dissociation (CID) was optimized at 12 eV under 1.0 mTorr of argon.

#### 2.4.5. Parental Interviews: Assessment of Clinical Effects of Vitamin E Supplementation

Questionnaires for parental interviews were created based on common behavioral changes observed in SLOS [8,26,27] and the Aberrant Behavior Checklist [28], with modifications. Questions were focused on changes (i.e., improvement or cessation of the symptoms) observed in (1) aggression and self-injury, (2) irritability (i.e., quick mood changes, sadness, inappropriate screaming, impatience, easy frustration, temper outbursts), (3) repetitive behavior (repetitive body movements), (4) hyperactivity/attention deficit and (5) sleep disturbance (difficulty falling asleep, fragmented sleep, early waking, reduced sleep duration, restless sleep). Parents were also questioned about whether amelioration was observed in skin photosensitivity and/or eczema. 

## 3. Results

### 3.1. Plasma Vitamin E and Vitamin A Status in SLOS Patients—Biochemical Effects of Vitamin E Supplementation

**Figure 2:**
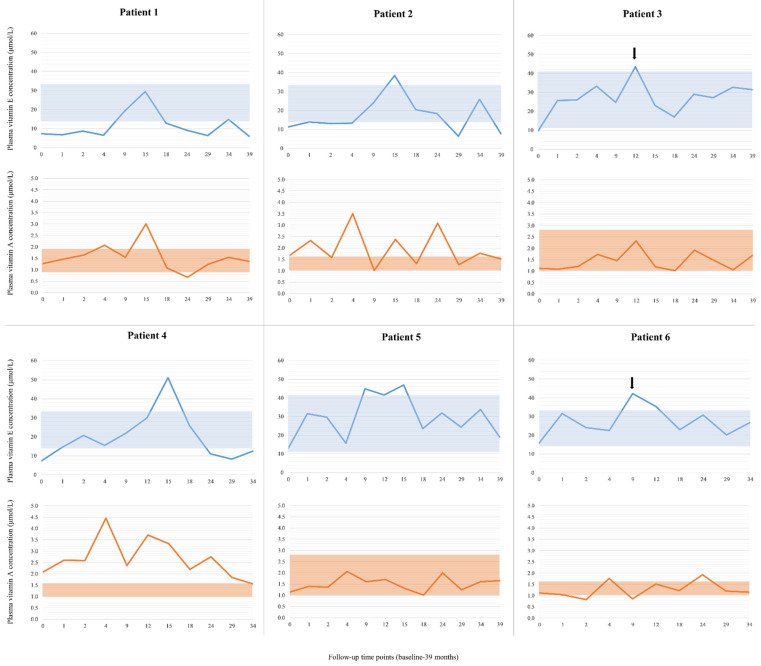
Plasma vitamin E and A concentrations in SLOS patients before and during supplementation with vitamin E. Age-matched reference intervals for plasma vitamin E and A concentration are shown by the blue and orange boxes, respectively. Starting doses of vitamin E were decreased by 50% in the case of Patients 3 and 6 (arrow). Time point “0” represents the baseline value.

In Figure 2, baseline (time point “0”) and follow-up (1–39 months) plasma vitamin A and E concentrations are shown for each patient, separately. Four patients (Patients 1, 2, 3 and 4) were vitamin E-deficient, while two had low–normal plasma vitamin E levels (Patients 5 and 6) at baseline. In four patients, vitamin E levels were increased by 2–2.7-fold of the baseline value after the first month, resulting in normalized vitamin E levels in two previously vitamin E-deficient patients (Patients 3 and 4) and a significant increase of plasma vitamin E level in two others (Patients 5 and 6) with low–normal baseline values. In two patients, who are brothers, normalization of the plasma vitamin E concentration was seen only later in the course of the treatment (Figure 2, Patients 1 and 2, at the ninth month of supplementation). Their parents reported irregular and inappropriate vitamin E intake because of frequent hospitalizations due to acute illnesses, which most likely led to insufficient supplementation. 

Plasma vitamin E concentration remained in the reference range at the majority of time points during the follow-up period in the case of three patients (Figure 2, Patients 3, 5 and 6). Patient 4 showed a very good response in the first 1.5 years of the study (Figure 2), then his plasma vitamin E concentration started to decrease in spite of appropriate vitamin intake. His mother reported that he developed daily, severe diarrhea. 

Since vitamin A is a fat-soluble vitamin, similarly to vitamin E, we also analyzed vitamin A levels. Individual measurements are shown in Figure 2 for each patient, separately. Plasma vitamin A concentrations were normal or even increased on average in the patients. In the case of Patient 4, plasma vitamin A concentrations were high at the first 4 follow-up time points, with a maximum of 4.45 µmol/L. The mother reported that the patient had been taking 3000 IU/day vitamin A (retinol) from birth. After stopping vitamin A supplementation, his results normalized very slowly.

### 3.2. Toxicity and Adverse Drug Reactions to Vitamin E Supplementation

We did not observe any obvious toxic effect during vitamin E supplementation. One patient developed diarrhea during the first 6 months of supplementation, most likely due to the oil vehicle, which resolved without intervention.

### 3.3. Behavioral Effects of Vitamin E Supplementation

Using questionnaires, we detected positive behavioral changes in three patients, and a summary of the clinical assessment is provided in Table 1.

### 3.4. Association of Clinical Response with Baseline 7-DHC + 8-DHC/Cholesterol Ratio

At baseline, plasma 7-DHC, 8-DHC and cholesterol levels were determined. In Table 2, molecular genetic data (genotypes only), vitamin E requirement, severity score, baseline plasma 7-DHC level, plasma 7-DHC + 8-DHC/cholesterol ratio and clinical response of the patients are summarized. 

Patients (*n* = 3; Patients 3, 5 and 6) who had low plasma (7-DHC + 8-DHC)/cholesterol ratio at baseline showed a clinical response to vitamin E supplementation, while in the case of patients (*n* = 3; Patients 1, 2 and 4) having a high baseline (7-DHC + 8-DHC)/cholesterol ratio, no clinical effect could be demonstrated (detailed in Table 1 and Table 2). It is also noteworthy that Patients 3 and 6 with the lowest ratio required less vitamin E to maintain normal plasma vitamin E levels compared to the others (Table 2). 

## 4. Discussion

Here, we report our observations with vitamin E supplementation in six SLOS patients. To the best of our knowledge, this is the first report of antioxidant treatment in this disease. 

One of our major findings at the onset of the study was that before vitamin E supplementation, plasma absolute vitamin E concentrations were either low or low–normal in the patients. Currently available data about vitamin E and/or fat-soluble vitamin status in SLOS patients is limited and contradictory. Rizzo et al. [29] reported severe vitamin E deficiency characterized by undetectable or very low plasma vitamin E levels, along with normal vitamin A status in SLOS patients. These authors suggested that vitamin E deficiency could be attributable to increased utilization of vitamin E rather than fat malabsorption. The fact that 7-DHC is prone to free radical oxidation is consistent with this suggestion since radical chain peroxidation would consume vitamin E, nature’s antioxidant. Indeed, the rate constant for the peroxidation of 7-DHC is an order of magnitude higher than that of any other lipid [30], making SLOS essentially an oxidative stress disorder. Significantly reduced plasma vitamin E concentrations were also detected in SLOS patients when compared to a control group by Haas et al., although this difference disappeared after vitamin E concentrations were adjusted to cholesterol levels [31]. Low plasma vitamin E concentrations were suggested to be the consequence of reduced lipoprotein carrier capacity in SLOS patients [31]. On the contrary, Elias et al. reported normal fat-soluble vitamin levels in six SLOS patients [32], and Kelley and Hennekam suggested that fat-soluble vitamin deficiency is not a common finding in SLOS [6]. 

Plasma vitamin E concentrations, as well as delivery to tissues, is dependent on lipid and lipoprotein metabolism. Cholesterol-adjusted vitamin E concentration is recommended for use in pathological conditions characterized by elevated (e.g., cholestatic liver disease) or decreased lipid concentrations (e.g., cystic fibrosis) [20]. However, in SLOS, reduced total sterol synthesis has been reported [33], and total sterol content of tissues in SLOS patients is comparable to that of controls [5]. Therefore, adjustment of plasma vitamin E concentration to cholesterol alone might not be appropriate in SLOS. Furthermore, there are no data available about the effect of cholesterol supplementation on plasma vitamin E levels in SLOS.

According to our follow-up data, supplementation with the oil-based vitamin E formula was successful in correcting, increasing and maintaining absolute plasma vitamin E concentrations in SLOS patients. Decline in plasma vitamin E level over time during supplementation could be attributed to two factors in our study: (1) inappropriate compliance/intake and (2) malabsorption due to severe, constant diarrhea unrelated to vitamin E supplementation. 

Patients received about 7–19-fold of the age/gender-matched recommended dietary allowance (RDA) without any signs of toxicity, i.e., bleeding symptoms. In this regard, it should be noted that the upper limit of daily intake was not exceeded in any instance [19]. One of the reasons for a higher need for vitamin E in SLOS could be inadequate absorption of the vitamin. Malabsorption cannot be ruled out, but based on available data, bile acid synthesis is not impaired in mild or moderately severe SLOS cases [33]. 

The normal or even elevated vitamin A levels we measured in our patients also support normal absorption of fat-soluble vitamins. In addition, in the case of Patient 4, having received vitamin A supplementation from birth, the vitamin A treatment had to be stopped because of very high plasma vitamin A values. Taken together, our data suggest that vitamin A supplementation is most likely not routinely needed for SLOS patients.

In addition to the laboratory findings, our preliminary clinical data further support the beneficial effects of vitamin E supplementation: 3 out of 6 SLOS patients showed a wide spectrum of behavioral improvements. These improvements encompassed reduced aggression, self-injury, irritability, hyperactivity/attention deficit, repetitive behavior and sleep disturbance (Patients 3, 5 and 6). Eczema was reported only in Patient 5, which resolved completely upon supplementation. Skin photosensitivity was reported in three patients and completely resolved in one of them (Patient 6). 

It should be noted that a positive clinical response to therapy was associated with a low baseline (7-DHC + 8-DHC)/cholesterol ratio, however, the notion that this parameter could predict the subset of SLOS patients who benefit from vitamin E supplementation deserves further investigation. One could speculate that for patients with high (7-DHC + 8-DHC)/cholesterol ratio, vitamin E cannot suppress all of the peroxidation that occurs, since the rate of oxidation depends on the local concentration of 7-DHC. This is consistent with the observation that the therapy is not successful with these patients (Table 2). The high levels of 7-DHC might overwhelm the antioxidant. We did not observe similar positive associations with severity score or age. 

7-DHC-derived oxysterols most likely influence normal brain development and function [14]. It has been suggested that cholesterol deficiency and/or precursor accumulation might influence behavior, including altered sleeping patterns [34,35]. Skin photosensitivity, a common dermatological finding in SLOS patients, might be the consequence of 7-DHC-derived oxysterols [36]. Vitamin E is a peroxyl radical scavenger and its main role has been claimed to protect long-chain polyunsaturated fatty acids in the cell membrane [37]. Based on the abovementioned data, observed clinical effects are most likely attributable to vitamin E lowering the concentration of 7-DHC-derived oxysterols, as shown before in vitro and in vivo [12].

It has to be noted that our study has its limitations. Only a small number of patients could be enrolled and there was no control group for ethical reasons because of the presumed beneficiary effect of vitamin E. A reversal study design would have further strengthened the power of the study. In spite of its shortcomings, we believe that our work clearly demonstrates that (i) vitamin E deficiency might be common in SLOS patients and it is most likely not attributable to malabsorption, and (ii) high-dose vitamin E supplementation can correct for the deficiency in these patients, probably caused by oxidative stress. Further clinical studies involving larger numbers of SLOS patients are needed to conclude a causal association between vitamin E supplementation and improvement of behavioral abnormalities and skin symptoms.

## 5. Conclusions

In summary, this report showed that the determination of vitamin E levels is warranted in SLOS patients, and vitamin E supplementation might be beneficial even in the absence of an overt deficiency. However, to achieve the full benefits of vitamin E administration, it is likely that vitamin E supplementation must start at young age, as it might be beneficial for the developing brain and body of SLOS patients, and it might potentially prevent or lessen the emergence of some SLOS-associated behavioral phenotypes.

## Figures and Tables

**Table 1:** Behavioral problems showing improvement following vitamin E administration.

SLOS-Associated Behavior and Photosensitivity ^a^	Patient 3	Patient 5	Patient 6
Sleep disturbance		+	+
Self-injury	+	+	+
Aggression		+	+
Repetitive body movements	+	+	+
Quick mood changes	+	+	+
Frequent sadness	+	+	
Inappropriate screaming			+
Temper outbursts	+	+	+
Attention deficit		+	+
Restlessness	+		
Uncontrollability	+		
Skin photosensitivity or eczema		+	+

^a^ Only symptoms that showed improvement in any of the patients are shown in the summary table, there were no behavioral changes recorded in Patients 1, 2 and 4. + = improvement.

**Table 2:** Summary of requirement and clinical effectiveness of vitamin E in relation to plasma 7-DHC + 8-DHC/cholesterol ratio.

Patient	Age ^a^	Gender	Genotype ^b^	Vitamin E (%RDA) ^c^	SS ^d^	7-DHC ^e^ (ng/uL)	7- + 8-DHC/C ^e^	Clinical Response ^f^
1	11 years	male	c.[964-1G>C];[1190C>T]	1882	50	70.7	4.9	no
2	5 years	male	c.[964-1G>C];[1190C>T]	1479	40	68.1	4.8	no
4	4 years	male	c.[976G>T];[452G>A]	1479	20	33.5	1.7	no
3	18 years	male	c.[964-1G>C];[1097G>T]	690	25	16.8	0.2	yes
5	21 years	female	c.[452G>A];[?]	1380	15	26.9	0.4	yes
6	5 years	male	c.[730G>A];[976G>T]	739	40	15.6	0.2	yes

^a^ Age at enrollment; ^b^ reference sequence (transcript): NM_001360.2, *DHCR7* gene sequencing was previously carried out in all patients [17,18]; ^c^ RDA, recommended dietary allowance of age- and gender-matched population (based on α-tocopherol equivalency [19]); ^d^ SS, severity score, determined according to Kelley and Hennekam [6], mild < 20, classical 20–50; ^e^ plasma 7-DHC (ng/µL), 8-DHC and cholesterol levels were measured before starting vitamin E supplementation; ^f^ detailed clinical response is provided in Table 1.

## Data Availability

All data generated or analyzed during this study are included in this published article.
